# Retraction: Clinical Performance and Survival Outcomes of All-on-4 Full-Arch Implant-Supported Prostheses in Patients With a Completely Edentulous Arch: A Retrospective Cohort Study

**DOI:** 10.7759/cureus.r264

**Published:** 2026-11-06

**Authors:** Jay Gohil, Mohammed Taslim Fathima, Dilip Chakravarthy, Sanpreet S Sachdev, Ankit Srivastava, Tejaswini K

**Affiliations:** 1 Department of Prosthodontics, K. M. Shah Dental College and Hospital, Sumandeep Vidyapeeth (Deemed to be University), Vadodara, IND; 2 Department of Oral and Maxillofacial Surgery, Care Dental College, Guntur, IND; 3 Department of Oral and Maxillofacial Surgery, Tirumala Institute of Dental Sciences and Research Center, Nizamabad, IND; 4 Department of Oral Pathology and Microbiology, Bharati Vidyapeeth (Deemed to be University) Dental College and Hospital, Navi Mumbai, IND; 5 Department of Prosthodontics, Seema Dental College and Hospital, Rishikesh, IND; 6 Department of Oral and Maxillofacial Surgery, Sri GGK Dental Clinic, Bellary, IND

This article has been retracted by the Editor-in-Chief due to concerns regarding the reliability of the reported data and the accuracy of the published methods and ethics statements. The underlying data were not available to verify the results. The majority of the authors disagree with the decision to retract.

